# Rapid molecular detection of respiratory pathogens in patients with bronchiectasis

**DOI:** 10.1183/23120541.01532-2025

**Published:** 2026-08-17

**Authors:** Daniela Alferes de Lima Headley, Migle Young, Rebecca C. Hull, Adam J. Bell, Chandani Hennayake, Rachel Galloway, Eve McIntosh, Zsofia Eke, Hollian Richardson, Merete B. Long, Sarah Williams-Macdonald, Edward Mountjoy, Marie-Jeanne H.C. Kempen, Declan Fawcett-Gibson, Daniel De Vega, James A. Long, Hazel Buchanan, Oriol Sibila, Stefano Aliberti, Rachel Dakin, Rebecca Holmes, Carolyn Clarke, James D. Chalmers

**Affiliations:** 1 Division of Respiratory Medicine and Gastroenterology, University of Dundee, Ninewells Hospital and Medical School, Dundee, UK; 2 LifeArc, Edinburgh, UK; 3 Pneumology Service, Hospital Clinic, IDIBAPS, CIBERES, University of Barcelona, Barcelona, Spain; 4 Department of Biomedical Sciences, Humanitas University, Milan, Italy; 5 University of Oxford, Radcliffe Department of Medicine, John Radcliffe Hospital, Oxford, UK

## Abstract

*To the Editor*:

## Abstract 2

**Bacterial quantification assays targeting rRNA for the detection of *H. influenzae, S. aureus, S. pneumoniae* and *M. catarrhalis* in bronchiectasis samples showed >90% sensitivity and consistently detected additional positives missed by the other methods** https://bit.ly/3PF437y

*To the Editor*:

Recurrent respiratory infections are a challenging aspect of bronchiectasis management [1, 2]. The most common pathogens detected are *Pseudomonas aeruginosa*, *Haemophilus influenzae*, Enterobacteriaceae, *Staphylococcus aureus*, *Streptococcus pneumoniae* and *Moraxella catarrhalis* [1]. Detection of pathogens from patient samples typically rely on culture and DNA based methods [3–5]. Culture is known to lack sensitivity, whilst DNA is thought to not correlate with cell viability [3, 4, 6, 7]. Ribosomal RNA (rRNA) is an alternative target as RNA is more likely to indicate viable bacteria, and RNA-based detection assays for mycobacteria have shown high sensitivity [8, 9]. We have previously developed and demonstrated the utility of a bacterial quantification assay (BQA) for *P. aeruginosa* with two patient cohorts, which showed higher sensitivity than culture and BioFire Film Array Pneumonia Plus Panel (a DNA- and RNA-based assay for bacterial detection) [10]. This work aims to develop and assess BQAs targeting rRNA for the detection of *H. influenzae, S. aureus, S. pneumoniae* and *M. catarrhalis* from bronchiectasis patient samples.

The EMBARC-BRIDGE patient cohort was used to address how detection of the pathogens using rRNA based qPCR (quantitative PCR) assays (BQAs) compared to culture, and other molecular based pathogen detection methods. The EMBARC-BRIDGE study is a multicentre prospective observational study integrated within the European Bronchiectasis Registry [1, 11, 12]. At the baseline visit, enrolled patients provided sputum samples for molecular microbiology analysis. All patients were clinically stable with no acute antibiotic treatment for at least 4 weeks prior to sampling. Where sample volume permitted, an additional portion from the same visit was submitted for routine clinical testing, allowing comparison between traditional culture results and those obtained through molecular assays and 16S rRNA gene sequencing. The BRIDGE study is registered under ClinicalTrials.gov ID: NCT03791086.

RNA was extracted from sputum using the ZymoBIOMICS RNA Miniprep kit (Zymo Research Catalogue Number R2001), according to manufacturers’ instructions with some modifications, including mechanical lysis and a 10-minute incubation step with lysostaphin. One-step real-time reverse-transcriptase qPCR (RT-qPCR) was performed to quantify the pathogen rRNA from patient sputum samples. Each reaction mixture contained 1× master mix, 1× reverse transcriptase enzyme, primers and a dual-labelled probe (Integrated DNA Technologies, Coralville, IA, USA) at optimised concentrations. For *S. aureus,* the forward primer (5′-AAGAACATATGTGTAAGTAACTG-3′) and reverse primer (5′-CTTTACGCCCAATAATTCC-3′) were used at 800 nM, with a probe (/56-FAM/ACCTAATCA/ZEN/GAAAGCCACGG/3IABkFQ/) at 400 nM. For *H. influenzae,* the forward primer (5′-CGCGTATTATCGGAAGAT-3′) and reverse primer (5′-CCACCAACTACCTAATCC-3′) were used at 400 nM, with a probe (/56-FAM/AGTGCGGGA/ZEN/CTGAGAGGC/3IABkFQ/) at 200 nM. For *M. catarrhalis,* the forward primer (5′-CTGATACTTAGTGGCGG-3′) and reverse primer (5′-GGTATTAGCTTGGGTTTCC-3′) were used at 400 nM, with a probe (/56-FAM/TGCTTAGGA/ZEN/ATCTGCCTAGTAGTGG/3IABkFQ/) at 200 nM. For *S. pneumoniae,* the forward primer (5′-AGGAGCTTGCTTCTCT-3′) and reverse primer (5′-ATCTGGTAGTGATGCAAG-3′) were used at 800 nM, with a probe (/56-FAM/CCGCATAAG/ZEN/AGTGGATGTTGC/3IABkFQ/) at 400 nM.

Reactions were backfilled with nuclease-free water for a total volume of 8 µL. For each reaction, 2 µL of a 1:10 dilution of the sample was included. All samples and controls were analysed in triplicate. The RT-qPCR protocol began with reverse transcription at 59°C for 10 min, followed by an initial denaturation at 95°C for 1 min. This was succeeded by 40 amplification cycles consisting of 95°C for 15 s and 60°C for 1 min, with fluorescence acquired. The upper limit of the 95% confidence interval of the limit of detection was used as the cut-off threshold for each assay; this was 12, 13, 14 and 17 copies per reaction for *S. aureus, H. influenzae, M. catarrhalis, and S. pneumoniae,* respectively. Following blank assessments to determine background amplification, a secondary cut-off of Cq<32 was applied for the *S. aureus* assay.

Multiplex PCR (BioFire Film Array Pneumonia Plus Panel) and synthetic full-length 16S rRNA gene LoopSeq were performed as previously described [10, 13, 14]. For BioFire and culture, all microorganisms detected were considered positive. Taxa, identified through 16S rRNA gene sequencing, were deemed as a positive result based on threshold of more than 10 reads or >0.1% relative abundance in a sample. Sequencing data have been submitted to European Nucleotide Archive (project accession number PRJEB88963).

The analytical performance of the assays was evaluated using adapted methods from relevant Clinical Laboratory Standards Institute standards, as previously described [10]. Sensitivity and specificity were determined as previously reported [15], and used the qualitative result of the BioFire as a reference. 95% confidence intervals were determined by percentile method using bootstrapping with 500 iterations.

This study included 111 samples from 98 patients. 46 of the 111 samples had multiple organisms detected by culture and/or qualitative molecular methods. 54 patients (55.1%) were male, with a mean age of 64.8 years (sd 14.1) and body mass index of 25.9 (sd 5.5). The mean forced expiratory volume in 1 s % predicted was 75.6% (sd 26.3). In the preceding year, 41 (41.8%) patients had experienced at least two exacerbations. Regarding underlying aetiology, 38 patients (38.8%) had idiopathic bronchiectasis, 15 (15.3%) post-infective bronchiectasis, 12 (12.2%) COPD, 9 (9.2%) primary ciliary dyskinesia, 7 (7.1%) asthma and 5 (5.1%) nontuberculous mycobacteria infection. Other causes of bronchiectasis were observed in less than four cases. All 111 samples underwent analysis with both BioFire and all BQAs; culture results were available for 57 samples and 16S rRNA gene sequencing data was available for 62.

All BQAs demonstrated suitable analytical performance (figure 1).

**FIGURE 1:**
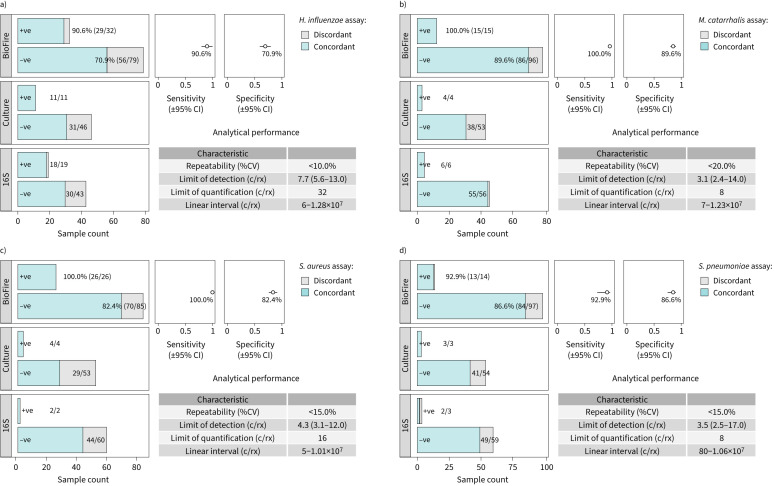
Sensitivity and specificity of the bacterial quantification assays. a) *Haemophilus influenzae*; b) *Moraxella catarrhalis*; c) *Staphylococcus aureus*; d) *Streptococcus pneumoniae*, when compared to the BioFire Pneumonia Plus Panel. Concordance/discordance results with culture and 16S ribosomal RNA gene sequencing status, and analytical performance. %CV: coefficient of variation; c/rx: copies per reaction.

The *H. influenzae* BQA (HI-BQA) demonstrated a sensitivity of 90.6% (29 of 32, 95% CI 80.0–100.0%) and a specificity of 70.9% (56 of 79, 95% CI 60.4–80.4%), indicating that the HI-BQA identified *H. influenzae* in 23 BioFire-negative samples. 11 out of 11 *H. influenzae* culture-positive samples were concordant with the HI-BQA. 31 out of 46 of the negative samples were concordant, indicating that the HI-BQA identified *H. influenzae* in 15 culture-negative samples. 18 of 19 *H. influenzae* 16S rRNA gene sequencing*-*positive samples were concordant with the HI-BQA, while 30 of 43 of the negative samples were concordant, indicating that the HI-BQA identified *H. influenzae* in 13 samples which were not identified by sequencing (figure 1a).

The *M. catarrhalis* BQA (MC-BQA) demonstrated a sensitivity of 100% (15 of 15, 95% CI 100.0–100.0%) and a specificity of 89.6% (86 of 96, 95% CI 83.5–95.0%), indicating that the MC-BQA identified *M. catarrhalis* in 10 BioFire-negative samples. 4 out of 4 *M. catarrhalis* culture-positive samples were concordant with the MC-BQA. 38 of 53 of the negative samples were concordant, indicating that the MC-BQA identified *M. catarrhalis* in 15 culture-negative samples. 6 out of 6 *M. catarrhalis* 16S rRNA gene sequencing-positive samples were concordant with the MC-BQA, while 55 out of 56 of the negative samples were concordant, indicating that the MC-BQA identified *M. catarrhalis* in one sample which was not identified by sequencing (figure 1b).

The *S. aureus* BQA (SA-BQA) demonstrated a sensitivity of 100% (26 of 26, 95% CI 100.0–100.0%) and a specificity of 82.4% (70 of 85, 95% CI 75.0–90.0%), indicating that the SA-BQA identified *S. aureus* in 19 BioFire-negative samples. 4 of 4 *S. aureus* culture-positive samples were concordant with the SA-BQA. 29 out of 53 of the negative samples were concordant, indicating that the SA-BQA identified *S. aureus* in 25 culture-negative samples. 2 of 2 *S. aureus* 16S rRNA gene sequencing*-*positive samples were concordant with the SA-BQA, while 44 out of 60 of the negative samples were concordant, indicating that the SA-BQA identified *S. aureus* in 16 samples which were not identified by sequencing (figure 1c).

The *S. pneumoniae* (SP-BQA) demonstrated a sensitivity of 92.9% (13 of 14, 95% CI 75.0–100.0%) and a specificity of 86.6% (84 of 97, 95% CI 79.6–93.3%), indicating that the SP-BQA identified *S. pneumoniae* in 13 BioFire-negative samples. 3 of 3 *S. pneumoniae* culture-positive samples were concordant with the SP-BQA. 41 out of 54 of the negative samples were concordant, indicating that the SP-BQA identified *S. pneumoniae* in 13 culture negative samples. 2 of 3 *S. pneumoniae* 16S rRNA sequencing*-*positive samples were concordant with the SP-BQA, while 49 out of 59 of the negative samples were concordant, indicating that the SP-BQA identified *S. pneumoniae* in 10 samples which were not identified by sequencing (figure 1d).

In summary, we report the initial assessment of rRNA-targeting BQAs for the detection of viable pathogens in bronchiectasis patient samples. Our aim was to assess the ability of these assays to accurately detect pathogens compared to established methods, including the BioFire FilmArray Pneumonia Plus Panel, 16S rRNA gene sequencing and traditional culture. Sensitivity and specificity were only calculated against BioFire due to limited numbers of positive samples from the other methods, though similar trends were observed with both culture and 16S rRNA gene sequencing. All BQAs showed high sensitivity (>90%) and consistently detected additional positives samples not identified by the other approaches. This most likely indicates increased sensitivity, although we cannot exclude the possibility of reduced specificity. A major challenge in the evaluation of novel assays like these is the absence of a definitive gold standard, especially since culture is known to lack sensitivity. However, extensive *in silico* analysis and testing with closely related bacterial species commonly found in human sputum, supports high specificity for the target organism. Future studies will be required to determine the clinical impact of the increased pathogenic detection observed with the BQA.
